# Impact of dietary fat levels and fatty acid composition on milk fat synthesis in sows at peak lactation

**DOI:** 10.1186/s40104-022-00815-y

**Published:** 2023-03-10

**Authors:** Li Zhe, Uffe Krogh, Charlotte Lauridsen, Mette Olaf Nielsen, Zhengfeng Fang, Peter Kappel Theil

**Affiliations:** 1 Key Laboratory for Animal Disease-Resistance Nutrition of China, Ministry of Education, Animal Nutrition Institute, Sichuan Agricultural University, 211 Huimin Road, Wenjiang District, Chengdu, 611130 China; 2 Department of Animal and Veterinary Sciences, Aarhus University, Foulum, Dk-8830 Tjele, Denmark

**Keywords:** Carbon metabolism, De novo fat synthesis, Dietary fatty acid, Fat balance, Mammary gene expression, Mammary lipogenesis, Milk fat production, Piglet growth

## Abstract

**Background:**

Dietary fat is important for energy provision and immune function of lactating sows and their progeny. However, knowledge on the impact of fat on mammary transcription of lipogenic genes, de novo fat synthesis, and milk fatty acid (FA) output is sparse in sows. This study aimed to evaluate impacts of dietary fat levels and FA composition on these traits in sows. Forty second-parity sows (Danish Landrace × Yorkshire) were assigned to 1 of 5 dietary treatments from d 108 of gestation until weaning (d 28 of lactation): low-fat control diet (3% added animal fat); or 1 of 4 high-fat diets with 8% added fat: coconut oil (CO), fish oil (FO), sunflower oil (SO), or 4% octanoic acid plus 4% FO (OFO). Three approaches were taken to estimate de novo milk fat synthesis from glucose and body fat.

**Results:**

Daily intake of FA was lowest in low-fat sows within fat levels (*P* < 0.01) and in OFO and FO sows within high-fat diets (*P* < 0.01). Daily milk outputs of fat, FA, energy, and FA-derived carbon reflected to a large extent the intake of those. On average, estimates for de novo fat synthesis were 82 or 194 g/d from glucose according to method 1 or 2 and 255 g de novo + mobilized FA/d according to method 3. The low-fat diet increased mammary *FAS* expression (*P* < 0.05) and de novo fat synthesis (method 1; *P* = 0.13) within fat levels. The OFO diet increased de novo fat synthesis (method 1; *P* < 0.05) and numerically upregulated mammary *FAS* expression compared to the other high-fat diets. Across diets, a daily intake of 440 g digestible FA minimized milk fat originating from glucose and mobilized body fat.

**Conclusions:**

Sows fed diets with low-fat or octanoic acid, through upregulating FAS expression, increased mammary de novo fat synthesis whereas the milk FA output remained low in sows fed the low-fat diet or high-fat OFO or FO diets, indicating that dietary FA intake, dietary fat level, and body fat mobilization in concert determine de novo fat synthesis, amount and profiles of FA in milk.

## Introduction

Milk production of sows is highly demanding and may account for 70% to 75% of the total requirements of energy and carbon at peak lactation [1–3]. Addition of dietary fat is common to achieve higher energy density in diets for lactating sows to increase the total energy intake and reduce the negative energy balance [4–6]. Milk fat is the major contributor to energy transfer from sows to their offspring and milk fat accounts for as much as 50% to 60% of that transfer [7]. But in spite of the importance of milk fat for the offspring, the impact of feed composition on de novo fat synthesis in the mammary gland is not well understood. Dietary fat increases milk fat output and besides that, triglycerides with different chain lengths and saturation of fatty acid (FA) may have distinct functions on immunity and antibacterial effects in animals [4, 8, 9]. After the hydrolysis of triglycerides, dietary medium-chain fatty acids (MCFA) are partly absorbed through the portal venous system and used either for β-oxidation or chain elongation, and they are also known to have antibacterial properties [9–12]. The long-chain fatty acids are absorbed and entering the circulation through the lymphatic system in the form of triglycerides incorporated in chylomicrons, and they can subsequently be taken up by the mammary gland and secreted in milk fat or used for oxidation [4, 13, 14]. Notably, the n-3 and n-6 long-chain polyunsaturated fatty acids (LC-PUFA) are essential FA for animals as they play a critical role in lipid metabolism, immune function, and cell division [10, 15, 16]. Considering the various roles of FA in the regulation of mammary metabolism, milk output, immune function, and health, research with lactating sows is needed to explore the effects of fat levels and FA composition on mammary fat synthesis to understand how milk FA profiles and daily FA output in milk is regulated in sow [4, 5, 17, 18]. To our knowledge, studies focusing on mammary de novo fat synthesis and the impact of dietary fat level and dietary FA composition is lacking for sows.

We hypothesized that sows consuming a diet high in fat or high in specific FA would increase either total or specific FA output in milk, regulate mammary expression of lipogenic, desaturating, and lactogenic genes, and affect milk fat synthesis. This study aimed to explore how dietary fat level (3% and 8% added fat) and FA composition (C8 to C24 with different degree of saturation) in late gestation and lactation sow diet affect milk FA profiles, mammary de novo fat synthesis, mammary gene expression, and progeny’s growth at peak lactation. In addition, three models based on different assumptions were developed to quantify mammary de novo fat synthesis from glucose, in an attempt to reveal how fat nutrition regulates milk fat synthesis. In this way knowledge gaps could potentially be identified regarding milk synthesis, which is much more technically challenging to study in sows due to the complicated anatomy of the mammary blood circulation compared to ruminants.

## Materials and methods

The experiment was conducted at the experimental facilities at the Department of Animal and Veterinary Sciences, Aarhus University, Foulum, Denmark, and experimental diets were produced at the university feed factory. All animal procedures complied with the Danish Ministry of Justice Law number 382 (10 June 1987), with Act number 726 (9 September 1993; as amended by Act No. 1081 of 20 December 1995).

### Animals, treatments and husbandry

Forty healthy second-parity sows (Danish Landrace × Yorkshire) were randomly assigned to 1 of 5 dietary treatments from d 108 of gestation to weaning (d 28 of lactation), and each treatment contained 8 replicates (sows). The dietary treatments included a low-fat control diet with 3% added animal fat and 4 high-fat diets containing 8% added fat from different fat sources; The fat sources represented MCFA (8% coconut oil; CO), n-3 LC-PUFA (8% fish oil; FO), a mix of a specific MCFA and LC-PUFA (4% octanoic acid + 4% FO; OFO), or n-6 LC-PUFA (8% sunflower oil; SO). The diets were formulated to meet the optimal supply of macronutrients relative to energy for sows according to Danish recommendations [19]. The feed ingredients and the analyzed chemical composition of the diets are shown in Table 1. The contents of dry matter (DM), crude protein, crude fat, starch, and gross energy (GE) in the diets were measured as described by Theil et al. [5].

**Table 1:** Diet composition and nutrients

Items	Low fat (3%)	Fat sources (8%)			
		**CO**	**OFO**	**FO**	**SO**
Ingredients, g/kg of feed					
Barley	387	328	328	328	328
Wheat	329	279	279	279	279
Soybean meal	223	278	278	278	278
Animal fat	30	-	-	-	-
Octanoic acid	-	-	40	-	-
Fish oil	-	-	40	80	-
Sunflower oil	-	-	-	-	80
Coconut oil	-	80	-	-	-
Monocalcium phosphate	10	12	12	12	12
Calcium carbonate	15	16	16	16	16
Sodium chloride	4	4	4	4	4
Mineral and vitamin mix^1^	2	2	2	2	2
Chemical composition (DM basis)^2^					
DM, %	89.9	89.8	87.1	90.3	90.2
Crude protein, %	18.3	19.8	20.8	20.0	20.3
Crude fat, %	7.2	10.5	10.6	10.3	10.0
Starch, %	44.6	38.8	38.6	40.1	39.2
GE, MJ/kg	18.8	19.6	20.2	19.5	19.5
ME, MJ/kg	16.0	17.0	17.5	16.8	17.0
Standard ileal digestible-Lysine, %	0.76	0.89	0.89	0.89	0.89
Standard ileal digestible-Methionine, %	0.23	0.25	0.25	0.25	0.25

*CO* Coconut oil, *OFO* Octanoic acid plus fish oil, *FO* Fish oil, *SO* Sunflower oil, *DM* Dry matter, *GE* Gross energy, *ME* Mmetabolizable energy

^1^ Provided per kilogram diet: Vitamin A 3.0 mg; Vitamin E 60 mg; Vitamin D_3_ 25 μg; Vitamin K 2.2 mg; Vitamin B_1_ 2.2 mg; Vitamin B_2_ 5.5 mg; Vitamin B_6_ 3.3. mg; *D*-pantothenic acid 16.5 mg; Niacin 22 mg; Folic acid 1.7 mg; Biotin 220 μg; Vitamin B_12_ 22 μg; Fe (FeSO_4_∙7 H_2_O) 150 mg; Cu (CuSO_4_∙5H_2_O) 20 mg; Zn (ZnO) 150 mg; Mn (MnO) 28 mg; I (KI) 0.34 mg; Se (Na_2_SeO_3_) 0.3 mg

^2^ The contents of DM, crude protein, crude fat, starch, and GE are analyzed value

Throughout this experiment, sows were kept in individual farrowing crates and fed one of the five diets twice daily (at 07:00 and 15:00 h, half of the daily meal each time). All sows were fed iso-energetically according to the Danish energy evaluation system from mating until d 2 of lactation, which is fairly similar to the net energy system [20]. With respect to metabolizable energy (ME), sows were fed 35 to 37 MJ ME/d (2.7 to 2.9 kg/d) from d 108 to 112 of gestation and 32 to 33 MJ ME/d (2.5 to 2.6 kg/d) from d 113 of gestation to d 2 of lactation. During lactation, sows were supplied 58 MJ ME/d (approximate 4.5 kg/d) from d 3 to 7, 77 MJ ME/d (approximate 6.0 kg/d) from d 8 to 13, and 90 to 103 MJ ME/d (7.0 to 8.0 kg/d) from d 14 until weaning at d 28 to ensure a high feed intake and minimal feed residues [4]. The litter size was standardized to 12 piglets by cross-fostering the day after parturition. Sows and piglets had free access to water throughout the experiment.

### Data and sample collection

Feed intake of sows was recorded daily, and the litter size and live weight of piglets were recorded weekly, from which the milk yield was estimated using the equations developed by Hansen et al. [7]. On d 10 and d 17 of lactation, both milk samples and mammary biopsies were collected 4 to 5 h after morning feeding, and milk samples were collected first, while the sows were held by snare restraint. The milk samples were collected after ear vein injection of 0.3 mL (10 IU/mL) oxytocin (Løvens Kemiske Fabrik, Ballerup, Denmark). The mammary biopsies were collected from three selected glands using a Manan Pro-Mag 2.2 biopsy gun with a 14-gauge needle (Medical Device Technologies, Gainesville, FL, USA) after washing, wiping with ethanol, and application of local anesthesia according to the method described by Theil et al. [21]. Approximately 20 mg biopsy was collected, immediately frozen in liquid nitrogen, and then transferred to −80 ℃ to store for later analysis of mRNA expression.

### Measurement of fatty acids in oils, diets, and milk

Fatty acids contents of oils, diets, and milk were measured by gas–liquid chromatography. Each oil source was measured once, the diets were measured in triplicate, and the milk samples were measured for an individual sow (*n* = 8/treatment). Fatty acids in oils, diets, and milk were extracted, saponified, and esterified from samples according to the method described previously [18, 22]. Briefly, for milk samples, 0.50 mL water, 2.00 mL methanol, and 1.00 mL chloroform were added to 500 mg of milk. The mixture was shaken for 1 min and then adding 1.00 mL water and 2.00 mL chloroform, and then shaken again and adding 1.00 mL water and 2.00 mL chloroform. The above mixture was shaken for 1 min and centrifuged at 1000 × *g* for 10 min. After which the chloroform (lower) phase was taken out and trans-esterified after saponification with NaOH and esterification with boron trifluoride methanol. The same method was used for oils and diets with the volumes of chloroform, methanol, and water kept in 1:2:0.8 before dilution. And then the FA methyl esters were determined by gas–liquid chromatograph (capillary) referenced to the method described by Rotenberg and Andersen [23].

### Measurement of milk composition

The contents of DM, fat, protein, and lactose in milk were measured using a MilkoScan FT2 instrument (Foss, Hillerød, Denmark), which was calibrated using bovine milk.

### Measurement of gene expression in mammary biopsies

Real-time reverse transcription-polymerase chain reaction (rRT-PCR) was used to quantify mammary gene expression as described by Theil et al. [21]. Briefly, total RNA was extracted from the frozen mammary biopsy after the tissue was homogenized with 350 μL RNeasy lysis buffer. The homogenate was diluted with 70% ethanol (1:1) before RNA was purified using a RNeasy mini kit (Qiagen, Albertslund, Denmark), and m-RNA was reverse-transcribed according to the manufacturer’s guide (Invitrogen, Taastrup, Denmark) using oligo-dT to synthesize cDNA. One microliter cDNA was amplified using gene-specific probes and primers with the TaqMan Universal PCR Master Mix (Applied Biosystems, Stockholm, Sweden) instrument. The signal was quantitatively detected using an ABI PRISM 7900 detection system (Applied Biosystems) to measure the labeled FAM (carboxyfluorescein) fluorophore on the 5′ end. Primer Express Version 2.0 software (Applied Biosystems) was used for primers and probe designs for all genes. Primer and probe sequences are shown in Table 2. Gene expression of *β-actin*, glyceraldehyde-3-phosphate dehydrogenase (*GAPDH*), fatty acid synthase (*FAS*), delta-6 desaturase (*D6D*), and α-lactalbumin (*α-LA*) were quantified, and both *β-actin* and *GAPDH* were found to be stable housekeeping genes. The difference in cycle threshold (Ct) value between the target gene and reference genes (i.e., ΔCt-values) was used for the statistical analysis, and the relative mRNA quantity was calculated by using the formula: Relative quantity = 2^−ΔΔCt^.

**Table 2:** Primer and probe sequences of target and housekeeping genes

Genes	Accession numbers	Primer/probe	Sequence (5′ to 3′)
*β-actin*	AY550069	Forward	ACCCAGATCATGTTCGAGACC
		Reverse	TCACCGGAGTCCATCACGAT
		Probe	CTGTATGCCTCTGGCCGCACCA
*GAPDH*	AF017079	Forward	GTCGGAGTGAACGGATTTGG
		Reverse	CAATGTCCACTTTGCCAGAGTTAA
		Probe	CGCCTGGTCACCAGGGCTGCT
*FAS*	AY954688	Forward	CGTGGGCTACAGCATGATAGG
		Reverse	GAGGAGCAGGCCGTGTCTAT
		Probe	CATCACCA
*D6D*	AY512561	Forward	GACGGCCTTCATCCTTGCT
		Reverse	ACAGAGAGATGGCCGTAATCGT
		Probe	CCTCTCAGGCCCAGGCTGGGTG
*α-LA*	M80520	Forward	ACAATGGCAGCACAGAATATGG
		Reverse	TCAGTAAGGTCATCATCCAGGAATT
		Probe	CTCTTCCAGATCAATAAT

*GAPDH* Glyceraldehyde-3-phosphate dehydrogenase, *FAS* Fatty acid synthase, *D6D* Delta-6 desaturase, *α-LA* α-lactalbumin

### Calculations

#### Output-input differences of fatty acids and carbon

The output-input differences of FA and carbon from FA between milk (output) and dietary digestible intake (input) were calculated based on feed intake, milk yield, and the measured FA composition in diets and milk samples. We assumed that the total tract digestibility of dietary FA was 85% based on the data from INRA [24] and previous studies [13, 25]. The FA output-input difference was calculated as follows:

$$\mathrm{Dietary}\;\mathrm{digestible}\;\mathrm{FA}\;\mathrm{intake}\;(\mathrm g/\mathrm d)=\mathrm{feed}\;\mathrm{intake}\;\left(\mathrm g/\mathrm d\right)\times\frac{\mathrm{dietary}\;\mathrm{individual}\;\mathrm{FA}\;\mathrm{content}\;\left(\%\right)}{100}\times\frac{85}{100}$$

$$\mathrm{FA}\;\mathrm{in}\;\mathrm{milk}\;(\mathrm g/\mathrm d)=\mathrm{milk}\;\mathrm{yield}\;\left(\mathrm g/\mathrm d\right)\times\frac{\mathrm{milk}\;\mathrm{individual}\;\mathrm{FA}\;\mathrm{content}\;(\%)}{100}$$

$$\mathrm{FA}\;\mathrm{output}-\mathrm{input}\;\mathrm{difference}\;(\mathrm g/\mathrm d)=\mathrm{FA}\;\mathrm{in}\;\mathrm{milk}\;(\mathrm g/\mathrm d)-\mathrm{dietary}\;\mathrm{digestible}\;\mathrm{FA}\;\mathrm{intake}\;(\mathrm g/\mathrm d)$$

Similar to the equations for FA difference, the output-input difference for FA-derived carbon between milk and dietary digestible intake was calculated as follows (assuming that digestibility of FA-derived carbon is 85%):

$$\mathrm{Carbon}\;\mathrm{in}\;\mathrm{dietary}\;\mathrm{digestible}\;\mathrm{FA}\;(\mathrm g/\mathrm d)=\mathrm{feed}\;\mathrm{intake}\;(\mathrm g/\mathrm d)\times\frac{\mathrm{dietary}\;\mathrm{individual}\;\mathrm{FA}\;\mathrm{content}\;\left(\%\right)}{100}\times\frac{12.01\;(\mathrm g/\mathrm{mol})\times\mathrm{carbon}\;\mathrm{number}\;\mathrm{in}\;\mathrm{individual}\;\mathrm{FA}\;\mathrm{in}\;\mathrm{diet}}{\mathrm{molecular}\;\mathrm{weight}\;\mathrm{of}\;\mathrm{individual}\;\mathrm{FA}\;(\mathrm g/\mathrm{mol})}\times\frac{85}{100}$$

$$\mathrm{Carbon}\;\mathrm{in}\;\mathrm{milk}\;\mathrm{FA}\;(\mathrm g/\mathrm d)=\mathrm{milk}\;\mathrm{yield}\;\left(\mathrm g/\mathrm d\right)\times\frac{\mathrm{milk}\;\mathrm{individual}\;\mathrm{FA}\;\mathrm{content}\;(\%)}{100}\times\frac{12.01\;(\mathrm g/\mathrm{mol})\times\mathrm{carbon}\;\mathrm{number}\;\mathrm{in}\;\mathrm{individual}\;\mathrm{FA}\;\mathrm{in}\;\mathrm{milk}}{\mathrm{molecular}\;\mathrm{of}\;\mathrm{individual}\;\mathrm{FA}\;(\mathrm g/\mathrm{mol})}$$

$$\mathrm{FA}-\mathrm{derived}\;\mathrm{carbon}\;\mathrm{output}-\mathrm{input}\;\mathrm{difference}\;(\mathrm g/\mathrm d)=\mathrm{carbon}\;\mathrm{in}\;\mathrm{milk}\;\mathrm{FA}\;(\mathrm g/\mathrm d)-\mathrm{carbon}\;\mathrm{indietary}\;\mathrm{digestible}\;\mathrm{FA}\;(\mathrm g/\mathrm d)$$

where the molecular weight of carbon is 12.01 g/mol. The total FA and total FA-derived carbon were the sum of individual value.

#### Estimation of de novo fat synthesis from glucose in mammary glands

The glucose taken up by mammary glands is mainly utilized for oxidation (energy and heat production), lactose synthesis, and de novo synthesis of milk fat [2]. Milk fat synthesized in the mammary gland of sows consists of fatty acids with an average chain length of 17 C-atoms esterified to glycerol in predominantly triglycerides [26]. Triacylglycerol accounts for approximately 90% on a w/w basis of milk fat [27]. The daily outputs of lactose and de novo fat synthesized from glucose was estimated using the following assumptions and equations. First, lactose output in milk (in mol/d) and glucose used for lactose synthesis were calculated as follows:

$$\mathrm{Lactose}\;\mathrm{output}\;\mathrm{in}\;\mathrm{milk}\;(\mathrm{mol}/\mathrm d)=\mathrm{milk}\;\mathrm{yield}\;(\mathrm g/\mathrm d)\times\frac{\mathrm{milk}\;\mathrm{lactose}\;(\%)}{100}\times\frac1{342\;(\mathrm g/\mathrm{mol})}$$

$$\mathrm{Glucose}\;\mathrm{used}\;\mathrm{for}\;\mathrm{lactose}\;\mathrm{synthesis}\;(\mathrm{mol}/\mathrm d)=2\times\mathrm{lactose}\;\mathrm{output}\;\mathrm{in}\;\mathrm{milk}\;(\mathrm{mol}/\mathrm d)$$

where, 342 g/mol is the molecular weight of lactose.

The total mammary uptake of glucose was calculated assuming that 36% of all glucose taken up by the mammary gland for a multiparous sow in peak lactation is used for lactose synthesis as found in a previous study based on C-balances across the mammary glands using multi-catheterized sows in a similar stage of lactation [2, 28]:

$$\mathrm{Mammary}\;\mathrm{glucose}\;\mathrm{uptake}\;\left(\mathrm{mol}/\mathrm d\right)=\frac{\mathrm{Glucose}\;\mathrm{used}\;\mathrm{for}\;\mathrm{lactose}\;\mathrm{synthesis}\;(\mathrm{mol}/\mathrm d)}{0.36}$$

**Fig. 1:**
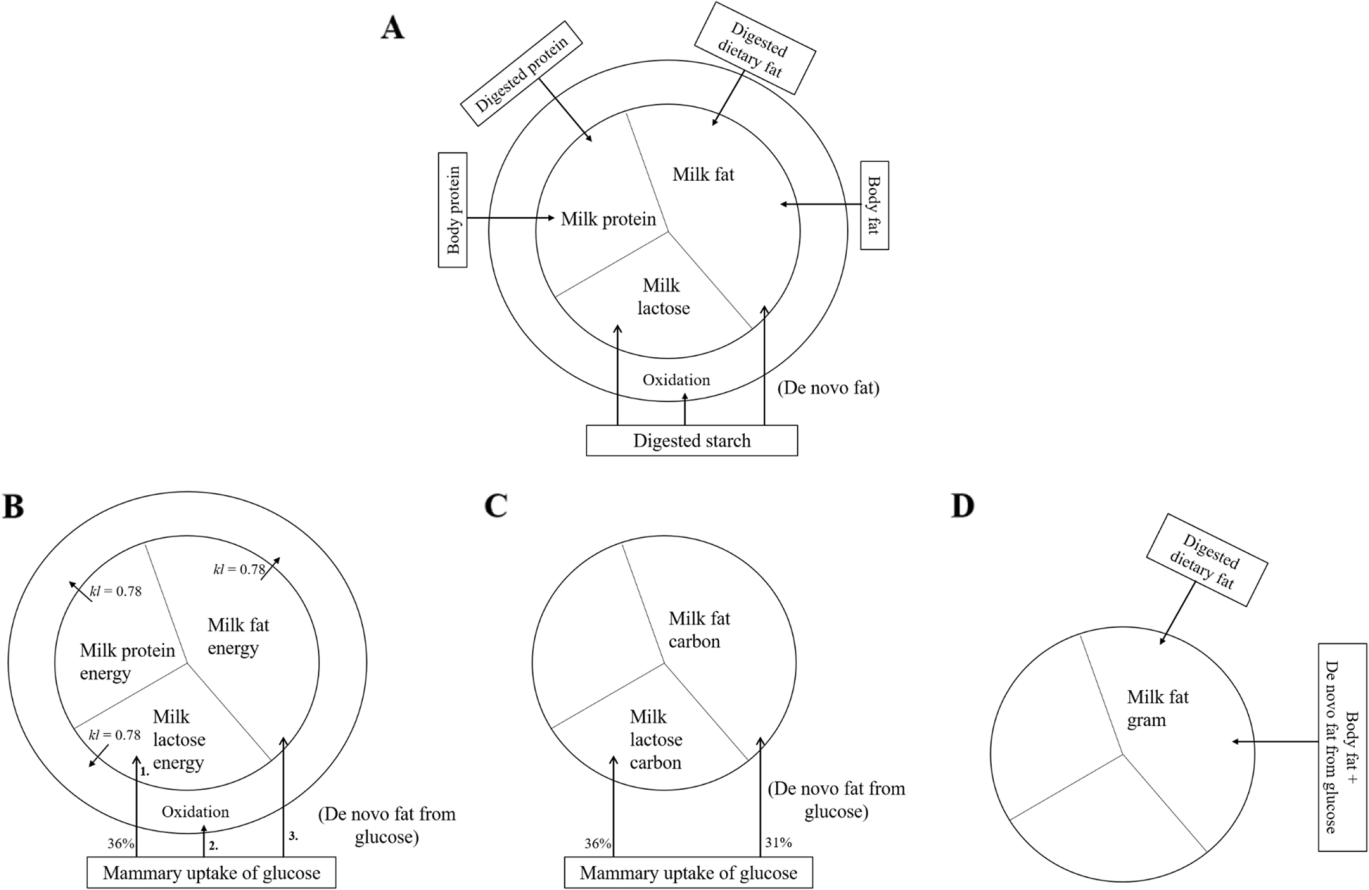
Prediction models of de novo synthesized fat from glucose. **A** Overall landscape of milk solids and precursors and approaches to quantify de novo fat synthesis from glucose (method 1 and 2; panel **B** and **C**) or fat from de novo synthesis + body fat (method 3; panel **D**). The outer circle denotes all the nutrients utilized for milk production including extra heat associated with milk synthesis, and the inner circle represents secreted milk solids; **B** Schematic presentation of prediction model for method 1, where the mammary glucose uptake was estimated by assuming that 36% of mammary glucose uptake is used for lactose synthesis, and mammary heat production is calculated by assuming that the energetic efficiency of milk production is 78%; **C** Schematic presentation of prediction model for method 2, where 36% of glucose carbon taken up by the mammary gland was used for lactose synthesis, and 31% of the glucose carbon taken up by the mammary gland was utilized for de novo fat synthesis; **D** Schematic presentation of approach to estimate endogenous FA from de novo synthesis from glucose plus body fat as evaluated from the input output difference

Subsequently, three different methods (Fig. 1B–D) were developed to predict the use of glucose for de novo fat synthesis. Method 1 and 2 were based on two different sets of assumptions from the literature regarding glucose partitioning between oxidation and de novo fat synthesis, whereas the third method quantified the endogenous FA contribution from de novo synthesized fat from glucose or body fat mobilization based on fatty acid balances.

**Method 1** estimated the amount of glucose used for de novo fat synthesis as the difference between the amount of glucose taken up by mammary glands minus that used for oxidation and lactose synthesis (Fig. 1B). The unknown in this method is the amount of glucose used for heat and energy production (oxidation), which was calculated based on the predicted GE content in milk [7] by assuming that the heat production is derived almost exclusively from oxidation of glucose in the mammary gland, while oxidation of protein and fat was considered negligible, and the energy efficiency of milk production was assumed to be 78% (*kl* = 0.78) as previously reported [29]. Thus,

$$\mathrm{Glucose}\;\mathrm{used}\;\mathrm{for}\;\mathrm{heat}\;\mathrm{production}\;\left(\mathrm{mol}/\mathrm d\right)=\left[\frac{\mathrm{GE}\;\mathrm{of}\;\mathrm{milk}\;\left(\mathrm{MJ}/\mathrm d\right)}{0.78}-\mathrm{GE}\;\mathrm{of}\;\mathrm{milk}\;\left(\mathrm{MJ}/\mathrm d\right)\right]/2.817\;(\mathrm{MJ}/\mathrm{mol})$$

where, 2.817 MJ/mol is the energetic value of glucose.

The amount of glucose used for de novo fat synthesis could then be calculated as follow:

$$\mathrm{Glucose}\;\mathrm{used}\;\mathrm{for}\;\mathrm{de}\;\mathrm{novo}\;\mathrm{fat}\;\mathrm{synthesis}\;(\mathrm{mol}/\mathrm d)=\mathrm{mammary}\;\mathrm{glucose}\;\mathrm{uptake}\;(\mathrm{mol}/\mathrm d)-\mathrm{glucose}\;\mathrm{used}\;\mathrm{for}\;\mathrm{heat}\;\mathrm{production}\;(\mathrm{mol}/\mathrm d)-\mathrm{glucose}\;\mathrm{used}\;\mathrm{for}\;\mathrm{lactose}\;\mathrm{synthesis}\;(\mathrm{mol}/\mathrm d)$$

**Method 2** was based on the assumption that 31% of glucose taken up by the mammary gland is used for de novo fat synthesis (Fig. 1C) as it has been found in a previous study [30]. The calculation was as follows:

$$\mathrm{Glucose}\;\mathrm{used}\;\mathrm{for}\;\mathrm{de}\;\mathrm{novo}\;\mathrm{fat}\;\mathrm{synthesis}\;(\mathrm{mol}/\mathrm d)=\mathrm{mammary}\;\mathrm{glucose}\;\mathrm{uptake}\;(\mathrm{mol}/\mathrm d)\times0.31$$

In both method 1 and 2, the de novo synthesis of fat considered to be predominantly milk triglycerides (in g/d) was then calculated from the amount of glucose available for de novo fat synthesis estimated using either method 1 or 2, and taking into account that 61% of carbons in this glucose is incorporated into the carbon skeleton of the de novo synthesized fat [31]. The de novo synthesized FAs in the sow mammary gland are primarily C16:0 and C16:1 with small amounts also of C12, C14, and (in contrast to ruminants) C18:0 and C18:1 [28]. We therefore additionally assumed the average chain length of de novo synthesized FA is 15.5 carbons, and with a carbon content of 3 in glycerol, a hypothetical triglyceride comprised of only de novo synthesized FA would have a molecular weight of 785 g/mol [32]. The calculation was as follows:

$$\mathrm{De}\;\mathrm{novo}\;\mathrm{fat}\;\mathrm{synthesis}\;\left(\mathrm g/\mathrm d\right)=\frac{\mathrm{glucose}\;\mathrm{used}\;\mathrm{for}\;\mathrm{de}\;\mathrm{novo}\;\mathrm{fat}\;\mathrm{synthesis}\;\left(\mathrm{mol}/\mathrm d\right)\times6\times0.61}{(15.5\times3\;\left(\mathrm{mol}\;\mathrm{FA}\;\mathrm{per}\;\mathrm{triglyceride}\right)+3)}\times785\;(\mathrm g/\mathrm{mol})$$

**Method 3** assumes that preformed FA from diets (input) are quantitatively taken up by the udder in sows in negative energy balance as at peak lactation. Subtracting the diet-derived FA input from the total output of FA in milk will then provide an estimate for the endogenous FA contribution, i.e. FA derived from either de novo synthesis from glucose within the mammary gland or from body fat mobilization (Fig. 1D).

$$\mathrm{Endogenous}\;\mathrm{FA}\;\mathrm{contribution}\;(\mathrm g/\mathrm d)=\mathrm{FA}\;\mathrm{output}-\mathrm{input}\;\mathrm{difference}\;(\mathrm g/\mathrm d)=\mathrm{FA}\;\mathrm{in}\;\mathrm{milk}\;(\mathrm g/\mathrm d)-\mathrm{dietary}\;\mathrm{digestible}\;\mathrm{FA}\;\mathrm{intake}\;(\mathrm g/\mathrm d)$$

### Statistical analysis

There were no significant differences between results from recordings or analyses from samples obtained on lactation d 10 and d 17, except for piglet performance. The mean of values observed for d 10 and d 17 were therefore used in the statistical analysis. All data were analyzed using the PROC MIXED procedure of SAS (version 9.4, SAS Institute Inc). For gene expression data, the delta Ct values (ΔCt = Ct of the target gene – mean Ct of the reference genes) of genes were used for statistical analysis. All the data were analyzed in 2 ways: as orthogonal contrasts between low-fat and the four high-fat diets, and by multiple comparisons between the four high-fat diets. Treatment differences between groups were determined using the PDIFF option, and the results were expressed as least-squares means with pooled-standard error, except for the genes relative abundance, which were reported as a mean ± 95% confidence limits. The CORR procedure of SAS was used to analyze the correlations of digestible FA intake and milk FA output, and the amounts of de novo synthesized fat using the two prediction methods. For the statistical evaluation, *P* ≤ 0.05 was declared as a significant response, while *P* ≤ 0.10 was declared as a tendency.

## Results

### Fatty acids composition in oils, diets, and milk

**Fig. 2:**
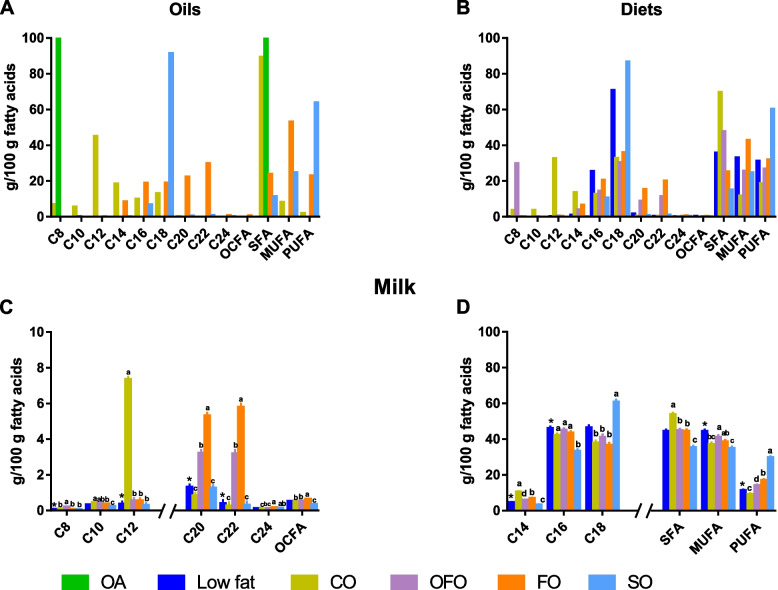
Fatty acids profiles in oils (**A**), diets (**B**), and milk (**C** and **D**). Each oil source was measured once, and the diets were measured triplicate, and the milk samples were measured for individual sow (*n* = 8/treatment). The significant difference between fat levels is marked with asterisk (*, *P* ≤ 0.05), meanwhile, the significant difference (*P* ≤ 0.05) among high-fat sources is marked with different small letters. OA = octanoic acid; CO = coconut oil; OFO = octanoic acid plus fish oil; FO = fish oil; SO = sunflower oil

The FA contents in the oils and whole diets are shown in Fig. 2A and B, respectively. The abundancy of individual FA in the whole diets reflected the composition of the fat source added to the specific diets: the CO diet was rich in saturated fatty acids (SFA; 69.6%), especially the medium-chain saturated fatty acids (MC-SFA) such as C8, C10, C12, and C14 (3.7%, 3.7%, 32.6%, and 13.7%); the OFO diet was rich in MC-SFA (47.7%) derived from the addition of C8 and the long-chain monounsaturated fatty acids (LC-MUFA; 25.5%) as well as LC-PUFA (26.8%) derived from FO; the FO diet was rich in LC-MUFA (42.8%) and LC-PUFA (31.9%); and the SO diet was rich in LC-PUFA (60.3%), especially C18:2n-6 (58.0%).

The FA composition in milk (Fig. 2C and D) showed that C16 and C18 were the most abundant FA across all diets, followed by C14, C20, and C22. Sows fed the low-fat diet had lower milk contents of C8, C12, C14, C20, C22, and poly-unsaturated fatty acids (PUFA), but higher milk contents of C16 and mono-unsaturated fatty acids (MUFA) as compared to sows fed the four high-fat diets (*P* < 0.01). As expected, sows fed the CO diet had the highest C10, C12, C14, and SFA in milk (*P* < 0.01). The highest proportion of C8 in milk was found in OFO sows (*P* < 0.01), while the highest C20, C22, and C24 were found in FO sows (*P* < 0.01). Sows fed the SO diet had the lowest C16, but highest C18 and PUFA content in milk (*P* < 0.01). In addition, the proportion of C20 and C22 in milk was lower in the SO and CO sows as compared to sows fed OFO and FO diets (*P* < 0.01).

### Sows’ nutrient intake, milk yield, milk outputs, and piglets’ growth

As shown in Table 3, dietary treatments had no impact on feed intake of sows, milk yield, or piglet growth (*P* > 0.05), but affected crude fat intake and ME intake (*P* < 0.05), and also tended to affect yield of milk components (*P* ≤ 0.10). Sow fed the low-fat control diet had, as expected, a lower daily crude fat intake (*P* < 0.05), ME intake (*P* < 0.05), and milk fat yield (*P* = 0.05) and tended to have a lower daily milk energy output (*P* = 0.10).

**Table 3:** Sows’ nutrient intake, milk yield and components output, and piglets’ growth rate

Items	Low fat (3%)	High fat (8%)^1^	Fat sources (8%)^2^				SEM	*P*-value^3^	
			**CO**	**OFO**	**FO**	**SO**		**Fat level**	**Fat source**
Feed intake, kg/d	7.04	7.18	7.37	7.07	7.22	7.04	0.145	0.39	0.25
DM intake, kg/d	6.33	6.41	6.62^a^	6.15^b^	6.52^a^	6.35^ab^	0.123	0.59	0.05
Crude fat intake, g/d	457^*^	665	697^a^	655^bc^	673^ab^	635^c^	12.4	< 0.001	< 0.01
ME intake, MJ/d	102^*^	110	112	108	110	108	2.2	< 0.01	0.32
Milk yield									
Day 10 to 17, kg/d	10.9	11.2	11.3	11.5	10.5	11.6	0.41	0.41	0.28
Day 1 to 28, kg/d	10.1	10.4	10.5	10.7	9.7	10.7	0.40	0.42	0.31
Daily output in milk									
DM, kg/d	1.99	2.12	2.22	2.05	1.98	2.23	0.083	0.13	0.09
Fat, kg/d	0.72^*^	0.81	0.92^a^	0.70^b^	0.73^b^	0.88^a^	0.043	0.05	< 0.01
Protein, kg/d	0.51	0.52	0.53	0.52	0.49	0.54	0.018	0.36	0.33
Lactose, kg/d	0.64	0.66	0.65	0.69	0.61	0.67	0.028	0.68	0.32
Energy, MJ/d	50.5	54.7	59.1^a^	51.0^b^	50.5^b^	58.1^a^	2.33	0.10	0.02
Piglet growth rate									
Litter size on d 1, pigs	12	12	12	12	12	12	-	-	-
Litter size on d 28, pigs	10.3	10.8	10.9	10.3	10.7	11.2	0.28	0.13	0.24
Litter average daily gain on d 1 to 28, kg/d	2.54	2.65	2.67	2.75	2.37	2.82	0.195	0.57	0.44

*CO* Coconut oil, *OFO* Octanoic acid plus fish oil, *FO* Fish oil, *SO* Sunflower oil, *DM* Dry matter

^1^ This column presents the mean value of high-fat groups from the contrast between low-fat group and four high-fat groups

^2^ The low-fat group is excluded from the statistical analysis of fat sources

^3^ Each treatment contained 8 replicates (*n* = 8)

^*^ Significant differences between fat levels are marked with an asterisk in the low-fat column (*P* ≤ 0.05)

^a–c^ Significant differences between the fat sources are marked with different superscripts (*P* ≤ 0.05)

Among the sows fed the high-fat diets, CO and FO sows had higher DM intake than the OFO sow (*P* = 0.05), while CO sows had higher daily crude fat intake than the OFO and SO sows (*P* < 0.05). Daily outputs of fat and energy in milk were lower for OFO and FO sows as compared to the SO and CO sows (*P* < 0.05), while sows fed the FO diet tended to have a lower daily output of DM in milk than the SO and CO fed sows (*P* = 0.09).

### Daily dietary intake and milk output of fatty acids and carbon from fatty acids

For the contrast between fat levels, sows consuming the low-fat control diet had greater daily intake of C16 (*P* < 0.01; Table 4), and decreased daily intake of the other grouped FA except for C18, total FA, and total FA-derived carbon (*P* < 0.01). In addition, the daily milk output of grouped FA was decreased except for C16, C18, SFA, and MUFA in the low-fat group (*P* < 0.10; Table 5). Among high-fat sources, the daily digestible intake and milk output of total FA and total FA-derived carbon were greater in the SO and CO groups compared with the OFO and FO groups (*P* < 0.01). Daily digestible intake and output of C8 was highest in the OFO group (*P* < 0.01), C10, C12, C14, and SFA were highest in the CO group (*P* < 0.01), and C18 and PUFA were highest in the SO group (*P* < 0.01). In addition, daily intake and output of C20 and C22 were highest in FO, intermediate in OFO, and lowest in SO and CO (*P* < 0.01). However, the daily output was not always in line with the daily digestible intake. For instance, although the highest daily digestible intake of C16 and MUFA were in the FO group (*P* < 0.01), there were no differences in the daily output of C16 and MUFA (*P* > 0.05). The FO and SO groups had a higher daily output of C24 than the other groups (*P* < 0.01) although the daily digestible intake of C24 in the SO group was lower than that in the OFO and FO groups (*P* < 0.01).

**Table 4:** Daily intake of digestible fatty acids and carbon from dietary fatty acids, g/d

Items	Low fat (3%)	High fat (8%)^1^	Fat sources (8%)^2^				SEM	*P*-value^3^	
			**CO**	**OFO**	**FO**	**SO**		**Fat level**	**Fat source**
C8	0.5^*^	36.7	18.7^b^	126.8^a^	0.8^c^	0.4^c^	1.07	< 0.001	< 0.001
C10	0.2^*^	4.9	18.8^a^	0.5^b^	0.1b^bc^	< 0.1^c^	0.11	< 0.001	< 0.001
C12	0.8^*^	42.5	167.0^a^	1.9^b^	0.9^b^	0.2^b^	1.00	< 0.001	< 0.001
C14	3.1^*^	29.0	69.8^a^	16.7^c^	28.5^b^	0.9^d^	0.52	< 0.001	< 0.001
C16	76.7^*^	66.3	64.0^b^	59.9^c^	89.4^a^	51.8^d^	1.52	< 0.001	< 0.001
C18	214.1	216.4	161.7^b^	122.2^c^	152.6^b^	428.9^a^	5.88	0.73	< 0.001
C20	4.9^*^	28.0	2.1^c^	37.5^b^	68.1^a^	4.2^c^	0.85	< 0.001	< 0.001
C22	1.4^*^	35.8	1.1^d^	48.6^b^	88.1^a^	5.5^c^	1.10	< 0.001	< 0.001
C24	0.4^*^	1.5	0.5^d^	1.5^b^	2.7^a^	1.3^c^	0.04	< 0.001	< 0.001
Odd-chain fatty acids	0.8^*^	0.9	0.4^d^	0.9^b^	1.6^a^	0.7^c^	0.02	< 0.001	< 0.001
SFA	108.3^*^	184.5	354.4^a^	200.5^b^	109.1^c^	74.0^d^	2.89	< 0.001	< 0.001
MUFA	99.7^*^	118.2	57.9^d^	106.3^c^	186.7^a^	121.7^b^	2.84	< 0.001	< 0.001
PUFA	94.9^*^	159.2	91.7^d^	109.8^c^	137.1^b^	298.1^a^	4.10	< 0.001	< 0.001
Total FA	303^*^	462	504^a^	417^b^	433^b^	494^a^	8.6	< 0.001	< 0.001
Total FA-derived carbon	224^*^	340	365^a^	299^c^	322^b^	374^a^	6.4	< 0.001	< 0.001

*CO* Coconut oil, *OFO* Octanoic acid plus fish oil, *FO* Fish oil, *SO* Sunflower oil, *SFA* Saturated fatty acids, *MUFA* Mono-unsaturated fatty acids, *PUFA* Poly-unsaturated fatty acids, *FA* Fatty acid

^1^ This column presents the mean value of high-fat groups from the contrast between low-fat group and four high-fat groups

^2^ The low-fat group is excluded from the statistical analysis of fat sources

^3^ Each treatment contained 8 replicates (*n* = 8)

^*^ Significant differences between fat levels are marked with an asterisk in the low-fat column (*P* ≤ 0.05)

^a–d^ Significant differences between the fat sources are marked with different superscripts (*P* ≤ 0.05)

**Table 5:** Daily output of fatty acids and carbon from fatty acids in milk, g/d

Items	Low fat (3%)	High fat (8%)^1^	Fat sources (8%)^2^				SEM	*P*-value^3^	
			**CO**	**OFO**	**FO**	**SO**		**Fat level**	**Fat source**
C8	0.3^*^	0.5	0.4^b^	1.1^a^	0.3^b^	0.2^b^	0.08	0.01	< 0.001
C10	1.9	2.4	3.3^a^	2.3^b^	2.1^b^	1.8^b^	0.22	0.06	0.001
C12	2.1^*^	16.6	57.7^a^	3.3^b^	3.1^b^	2.1^b^	2.30	< 0.001	< 0.001
C14	28.9^*^	47.9	83.2^a^	37.2^bc^	43.8^b^	27.2^c^	3.63	< 0.001	< 0.001
C16	292.5	289.4	329.1	286.6	272	269.9	19.18	0.88	0.12
C18	299.7	317.7	292.6^b^	258.0^b^	233.8^b^	486.4^a^	21.53	0.44	< 0.001
C20	8.2^*^	17.5	6.5^c^	20.2^b^	33.1^a^	10.0^c^	1.10	< 0.001	< 0.001
C22	2.2^*^	15.0	1.9^d^	20.0^b^	35.8^a^	2.4^c^	1.12	< 0.001	< 0.001
C24	0.6	0.7	0.5^b^	0.6^b^	0.9^a^	0.9^a^	0.09	0.08	0.01
Odd-chain fatty acids	3.1	3.3	3.6	3.2	3.7	2.6	0.30	0.56	0.07
SFA	282.4	317.1	419.5^a^	284.3^b^	278.5^b^	286.0^b^	19.86	0.11	< 0.001
MUFA	284.9	269.5	291.2	262.3	244.7	279.6	19.68	0.46	0.33
PUFA	73.0^*^	125.4	69.3^c^	86.8^bc^	106.2^b^	239.3^a^	8.08	< 0.001	< 0.001
Total FA	638	696	778^a^	613^b^	593^b^	802^a^	41.4	0.19	0.002
Total FA-derived carbon	485	539	587^a^	480^b^	478^b^	612^a^	31.5	0.11	0.01

*CO* Coconut oil, *OFO* Octanoic acid plus fish oil, *FO* Fish oil, *SO* Sunflower oil, *SFA* Saturated fatty acids, *MUFA* Mono-unsaturated fatty acids, *PUFA* Poly-unsaturated fatty acids, *FA* Fatty acid

^1^ This column presents the mean value of high-fat groups from the contrast between low-fat group and four high-fat groups

^2^ The low-fat group is excluded from the statistical analysis of fat sources

^3^ Each treatment contained 8 replicates (*n* = 8)

^*^ Significant differences between fat levels are marked with an asterisk in the low-fat column (*P* ≤ 0.05)

^a–c^ significant differences between the fat sources are marked with different superscripts (*P* ≤ 0.05)

### Correlations between digestible fatty acids intake and milk fatty acids output

**Fig. 3:**
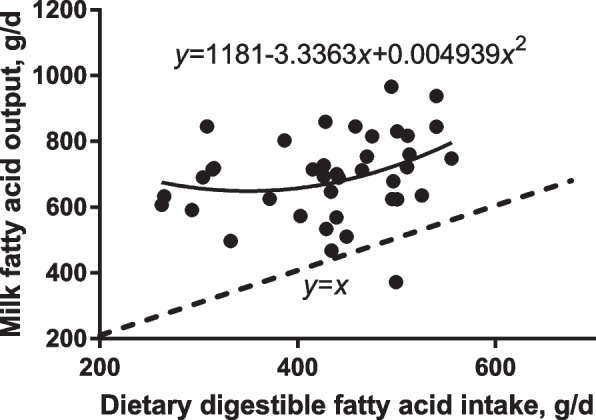
Correlation between daily dietary digestible fatty acids intake and daily milk fatty acids output. The dash line represents *y* = *x*, indicating no FA from de novo synthesis from glucose and from mobilized body fat

The relationship between intake of digestible FA and milk FA output (Fig. 3) revealed that the milk FA output always exceeded the intake, while the difference between digested FA and FA in milk output reflects the fat synthesized from endogenously derived FA, i.e., de novo synthesized or derived from body fat mobilization (method 3). The milk FA output was curvilinearly related to the daily intake of digestible FA across all sows (Fig. 3). The lowest FA output in milk was achieved when the digestible FA intake was 338 g/d, where the FA output amounted to 618 g/d and the FA of endogenous origin amounted to 280 g/d. The lowest difference between digestible FA intake and milk FA output (229 g/d) was achieved when digestible FA intake amounted to 440 g/d, whereby 669 g/d of FA was secreted in milk and the milk FA yield increased with daily intake of digestible FA exceeding 440 g.

### Mammary synthesis of fatty acids and carbon in milk (method 3)

As shown in Table 6, C14, C16, and C18 were the top 3 grouped FA with greatest output-input difference, and both SFA and MUFA had positive output-input differences, whereas C8 and PUFA had negative output-input differences. Between different fat levels, sows fed the low-fat diet had higher output-input differences for C8, C10, C12, C20, C22, C24, and total FA (*P* < 0.01), and tended to have higher output-input differences for C14 (*P* = 0.09), SFA (*P* = 0.06), and total FA-derived carbon (*P* = 0.08) as compared to the sows fed high-fat diets.

**Table 6:** The output-input difference of fatty acids and carbon from fatty acids, g/d

Items	Low fat (3%)	High fat (8%)^1^	Fat sources (8%)^2^				SEM	*P*-value^3^	
			**CO**	**OFO**	**FO**	**SO**		**Fat level**	**Fat source**
C8	-0.2^*^	-36.1	-18.2^b^	-125.6^c^	-0.6^a^	-0.1^a^	1.09	< 0.001	< 0.001
C10	1.7^*^	-2.5	-15.5^b^	1.9^a^	2.0^a^	1.8^a^	0.26	< 0.001	< 0.001
C12	1.4^*^	-26.0	-109.3^b^	1.3^a^	2.0^a^	1.9^a^	2.91	< 0.001	< 0.001
C14	25.8	18.9	13.3^b^	20.5^ab^	15.4^ab^	26.3^a^	3.79	0.09	0.12
C16	215.9	223.2	265.1^a^	226.6^ab^	182.9^b^	218.1^ab^	19.16	0.72	0.04
C18	85.5	101.3	131.0^a^	135.8^a^	80.7^ab^	57.6^b^	22.35	0.51	0.06
C20	3.3^*^	-10.4	4.4^a^	-17.3^b^	-34.5^c^	5.8^a^	1.32	< 0.001	< 0.001
C22	0.8^*^	-20.7	0.7^a^	-28.7^c^	-51.7^d^	-3.1^b^	1.04	< 0.001	< 0.001
C24	0.2^*^	-0.8	≈0.1^a^	-0.9^c^	-1.8^d^	-0.4^b^	0.08	< 0.001	< 0.001
Odd-chain fatty acids	2.3	2.4	3.3^a^	2.3^b^	2.1^b^	1.9^b^	0.30	0.85	0.01
SFA	174.1	132.6	65.1^b^	83.8^b^	169.4^a^	212.0^a^	20.18	0.06	< 0.001
MUFA	185.2	151.5	233.3^a^	156.0^b^	58.8^c^	157.9^b^	20.46	0.13	< 0.001
PUFA	-21.8	-33.8	-22.5^a^	-22.9^a^	-30.8^a^	-58.8^b^	8.38	0.18	0.02
Total FA (endogenous FA)	335^*^	235	274	196	161	308	42.5	0.03	0.08
Total FA-derived carbon	262	200	222	182	157	239	32.2	0.08	0.29

*CO* Coconut oil, *OFO* Octanoic acid plus fish oil, *FO* Fish oil, *SO* Sunflower oil, *SFA* Saturated fatty acids, *MUFA* Mono-unsaturated fatty acids, *PUFA* Poly-unsaturated fatty acids, *FA* Fatty acid

^1^ This column presents the mean value of high-fat groups from the contrast between low-fat group and four high-fat groups

^2^ The low-fat group is excluded from the statistical analysis of fat sources

^3^ Each treatment contained 8 replicates (*n* = 8)

^*^ Significant differences between fat levels are marked with an asterisk in the low-fat column (*P* ≤ 0.05)

^a–d^ Significant differences between the fat sources are marked with different superscripts (*P* ≤ 0.05)

For the analysis of the sows fed the high-fat diets, the output-input difference of C8 was lowest in the OFO group (*P* < 0.01) and sub-lower in the CO group as compared with the other groups (*P* < 0.01). The output-input difference of C10 and C12 was only negative in the CO group (*P* < 0.01), C16 was highest in CO group and smallest in FO group (*P* < 0.05), C18 was higher in the OFO and CO groups as compared with the SO group (*P* = 0.06), and C20, C22, and C24 were lowest in the FO group and sub-lower in the OFO group (*P* < 0.01). In addition, the output-input difference of SFA was higher in the FO and SO groups than the OFO and CO groups (*P* < 0.01), PUFA was lowest in the SO group (*P* < 0.01), total FA was higher in the SO and CO groups than the FO group (*P* = 0.08). The output-input difference of MUFA was highest in the CO group, lowest in the FO group, and intermediate in the OFO and SO groups (*P* < 0.01).

### De novo fat synthesis in mammary gland and correlations between different prediction methods

**Fig. 4:**
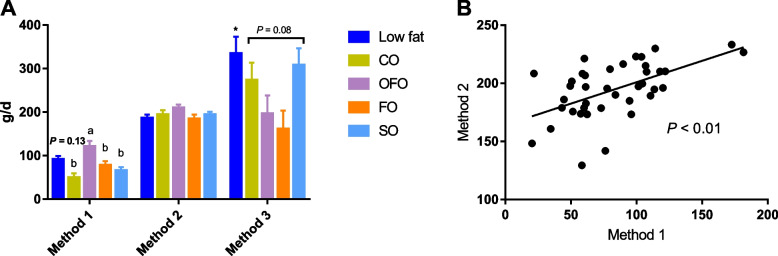
De novo fat synthesis from glucose and synthesized FA. Panel **A** Methods 1 and 2 estimate the mammary de novo synthesis of fat from glucose, method 3 estimates the mammary endogenous FA from glucose plus mobilized body fat. Each treatment contained 8 replicates (*n* = 8). The significant difference between fat levels is marked with asterisk (^*^P ≤ 0.05), while significant differences (*P* ≤ 0.05) among high-fat diets are marked with different small letter. CO = coconut oil; OFO = octanoic acid plus fish oil; FO = fish oil; SO = sunflower oil. Panel **B** shows the correlation between de novo synthesis of fat quantified using method 1 and 2

Results showed that the predictions of de novo fat synthesis from glucose by method 1 and 2 were positively related (*P* < 0.01, *r* = 0.30; Fig. 4B). The prediction method 1 suggested that sows fed the low-fat diet had the numerically highest de novo fat synthesis (*P* = 0.13; Fig. 4A); among different high-fat sources, the OFO group increased the amount of de novo synthesized fat compared with the SO and CO groups (*P* < 0.05). However, there was no significant diet effect on de novo synthesis of fat using prediction method 2, neither with respect to fat levels nor high-fat sources (*P* > 0.05; Fig. 4A). In addition, method 3 indicated that sows fed the low-fat diet had increased mammary synthesis of fat from endogenously derived FA as compared with the high-fat groups (*P* < 0.05; Fig. 4A), while the SO and CO groups relied more on endogenously derived FA for milk fat synthesis than the FO group within high-fat sources (*P* = 0.08).

### Mammary gene expression

**Fig. 5:**
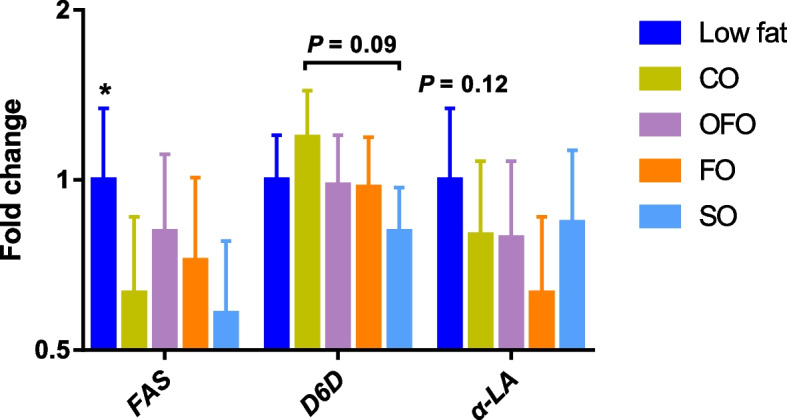
Gene expression in the mammary gland. Each treatment contained 8 replicates (*n* = 8). The significant difference between fat levels is marked with asterisk (^*^P ≤ 0.05), meanwhile, the significant difference (*P* ≤ 0.05) between high-fat sources is marked with different small letter. CO = coconut oil; OFO = octanoic acid plus fish oil; FO = fish oil; SO = sunflower oil; *FAS* = fatty acid synthase; *D6D* = delta-6 desaturase; *α-LA* = α-lactalbumin

As shown in Fig. 5, the relative mRNA abundance of *FAS* was lower (*P* < 0.05), and that of *α-LA* was numerically lower (*P* = 0.12) when sows were fed diets containing high-fat (8% added fat) as compared with the low-fat diet with 3% added fat. Among different high-fat sources, the mRNA abundance of *D6D* tended to be lower in the SO sows compared with the CO sows (*P* = 0.09).

## Discussion

In this study, feeding peak lactation sows different fat levels (3% vs. 8% added fat) or different fat sources in the high-fat diets had no influence on overall milk yield, which is consistent with previous studies. Thus, sows fed a diet without any added fat or diets with 8% added from either animal fat, FO, SO, or CO had similar milk yields [4], and in a dose–response feeding trial there was no impact of increasing dietary soya oil (0, 3.3%, 6.6%, and 9.9%) on milk yield [6]. Although overall milk yield was unaffected by fat supplementation, the present study showed that the daily fat output was lower in sows fed the low-fat control diet as compared with sows fed the high fat diets. Furthermore, the daily milk outputs of fat and energy were highest in sows fed the SO and CO diets as compared with sows fed the OFO and FO diets, which could not be explained by differences in the crude fat intake but the FA intake. In support, a previous study showed that when the added fat in sow diets was increased from 0 to 8%, the daily milk fat output increased depending on the fat source. In the same study, a CO diet showed a greater daily fat output as compared with sows fed FO and SO diets [4]. A review by Rosero et al. [33] indicated that sows fed fat rich diets had enhanced milk fat output and increased piglet growth. However, piglet growth was not affected in our study, neither by dietary fat levels nor by dietary fat sources in sow diets with 8% added fat, and this was despite of differences in milk fat and milk energy outputs. This is most likely because early piglet growth is mainly driven by protein and water retention [34]. That dietary fat intake and milk fat output are not major determinants for piglet growth are confirmed by several studies. Neal et al. [35] observed that piglets’ growth was unaffected by increases in fat levels in the sow diet from 3% to 6% or even 9%. Sows fed with 8% added SO were reported to have higher litter weight gain than sows fed 8% added FO even though the milk fat output was similar [4]. Furthermore, although milk yield and milk composition did not change, sows fed a diet with only 3% added fat showed lower litter weight gain when compared with 6% and 9% added fat [6]. The abovementioned studies support findings from our study that milk fat output and milk fat composition can be regulated by fat level and FA composition in sow diets (i.e., fat sources), but it is important to stress that milk fat output does not seem to be a major determinant of piglet growth.

In the current study, the low-fat diet resulted in lower daily intake and less milk output of even chain FA as compared with sows fed the high fat diets, except for intake of C16 and C18 FA. This was expected because of the specific FA enrichment in the different high-fat diets. For sows fed OFO, as much as 99.1% of the daily digested amounts of C8 was metabolized, most likely by the mammary glands and other organs. Octanoic acid is easily oxidized [2], but most likely it was partly used as a precursor for de novo FA synthesis because the carbon difference in digested FA intake between OFO and FO disappeared in milk FA. The OFO diet increased C14 but not C10 + C12 in milk compared to that in the FO diet, indicating that sow mammary gland is less prone to synthesize FA with a chain length shorter than C14, and the C8 + C10 + C12 in milk are likely of dietary origin. However, the majority of digestible C8 (97.3%), C10 (82.4%), and C12 (65.4%) in the CO group must have been metabolized elsewhere in the sow, while the output-input difference of C14 between dietary intake and milk output was the lowest. When sows were fed the SO diet, their intake of C18 was doubled, and their milk output of C18 increased in turn by 1.7- to 2.1-fold as compared with the other dietary groups, but the output-input difference was smaller on the SO diet than on the other diets, indicating a down-regulation of C18 use for milk fat synthesis. Notably, results from OFO and FO fed sows indicated that the daily disappearance (output-input difference) of C20 (4.3 g/d), C22 (6.5 to 7.2 g/d), and C24 (0.2 g/d) was constant for each percent-unit of FO added to the diet (4% in OFO diet and 8% in FO diet). In addition, the higher ingestion of MUFA in FO, PUFA in SO, and SFA in CO illustrated a higher disappearance or a lower mammary synthesis rate of those FA in milk over diet. In summary, the specific enriched FA in diets increased their daily dietary intake and output in milk, and illustrated a higher disappearance or a lower mammary synthesis in milk.

The number of carbon atoms quantified as the difference between milk FA output of carbon and the intake of digestible carbon, represents to a great extent the energy metabolism occurring in the mammary gland with respect to de novo fat synthesis [3]. Our results showed that sows fed the low-fat diet had lower daily intake of FA and FA-derived carbon, while these sows only secreted numerically less FA and FA-derived carbon through milk, which resulted in a higher output-input difference of carbon between milk output and digestible intake. In high-fat diets, the intake and output of FA and FA-derived carbon were lower in the OFO and FO groups than in the SO and CO groups, while the lowest output-input difference of FA was observed in the FO group. The FA and FA-derived carbon output in milk were consistent with the changes in milk fat and energy output in the present study. We further found that the milk FA output curvilinearly changed as the dietary digestible FA intake increased, and the lowest difference (229 g/d) was achieved when the intake of digestible FA was 440 g/d indicating that the lowest contribution of endogenous FA to milk fat synthesis from either de novo synthesis from glucose or from mobilized fat (229 g/d) was achieved. Thus, if sows had a dietary intake of digestible FA below 440 g/d, sows increase their milk FA from either de novo FA synthesis (depending on substrate availability) or body fat mobilization to support a daily milk FA output of approximately 669 g/d; whereas if sows ingest more than 440 g/d of digestible FA, their daily output of milk FA increases as the intake of digestible FA exceeds 440 g/d.

Milk fat originates from three sources namely the from dietary fat, body fat mobilization, and de novo fat synthesis in the mammary gland [27]. Glucose is the quantitatively most important substrate for de novo fatty acid synthesis in sows [2, 30] and it is also used for synthesis of the glycerol backbone to which FA is esterified, whether de novo synthesized or derived from body fat mobilization. Hence, two models were developed to predict the overall de novo synthesis of fat from glucose by the mammary gland, using different assumptions, and a third method was developed to compare these values with the fat being synthesized from both glucose and mobilized body fat. According to method 1, sows fed the low-fat diet had numerically greater de novo synthesized fat than sows fed high-fat; and for sows fed high-fat diets, the OFO diet had the greatest amount of de novo synthesized fat, although the crude fat intake in this group was similar to the FO group and higher than the SO group. The high de novo fat synthesis in the OFO group was most likely due to the high proportion of C8 in this diet. One disadvantage of method 1 is that a constant energetic efficiency of milk production (*kl* = 0.78) was assumed [5]. The *kl* for de novo synthesized fat from glucose is expected to be approximately 0.67 because only 4 out of 6 carbons are used for milk fat synthesis while two carbons are lost as CO_2_ [3], and finally, only 61% of carbons in glucose are incorporated into the carbon skeleton of the de novo synthesized fat [31]. In contrast, if fat synthesis is solely using preformed FA from either mobilized body fat or dietary fat, we would expect an energetic efficiency of 0.89 [36]. Thus, if equal amount of milk fat is synthesized from glucose and body fat, it is reasonable to assume a *kl* of 0.78, but if more fat is synthesized from glucose, the *kl* should be lower and vice versa higher if most milk fat was synthesized from mobilized FA. Method 1 may therefore have overestimated the amount of de novo synthesized fat in the high-fat OFO fed sows, because a greater amount of their de novo fat synthesis originated from glucose than that from body mobilization. In method 2, it was assumed that a constant proportion of the glucose taken up by the mammary gland would be used for de novo fat synthesis [30], which in turn resulted in estimates of de novo synthesized fat that were similar irrespectively of the diet. This is unlikely to be correct, as increased mammary uptake of long-chained fatty acids most probably would down-regulate de novo fatty acid synthesis from glucose. Although the estimates for de novo synthesized fat by method 1 (range 50 to 123 g/d) and method 2 (range 185 to 210 g/d) were positively related to each other, we therefore consider results from method 1 to be more reliable. According to the difference between method 1 and method 3, it seems likely that the SO and CO groups had greater fat mobilization than the OFO and FO groups, and so did sows fed the low-fat diet as compared with the high-fat diets. Despite discrepancies between results obtained with the 3 different methods, the modeling generally suggests that both fat supplementation (3% vs. 8% added fat) and FA profiles in sow diets are important for de novo synthesis of fat in the mammary gland, and the digestible intake and milk output of FA interact with body fat mobilization.

The *FAS* plays an important role in the process of de novo FA synthesis [37, 38] and is easily suppressed by high dietary levels of fat and unsaturated FA [39]. The contrast between fat levels showed that the mammary expression of *FAS* gene was greater when sows were fed the low-fat diet, which is most likely due to the low dietary fat level and a reduced feed-back inhibition on de novo FA synthesis from preformed long chain FA. This result was consistent with the positive relationship between de novo FA in milk and mammary *FAS* expression [40] and in line with the decreased mammary *FAS* expression in dairy cows fed increasing dietary crushed sunflower seed [39], and this then suggests that the prediction method 1 is more reliable than method 2, which assumes a constant fraction of mammary glucose uptake is used for de novo fat synthesis. Although differences in FA composition of the high-fat sources did not affect mammary *FAS* expression, the *FAS* expression was numerical greater when sows were fed the OFO diet, which suggest that the de novo fat synthesis could have been increased. Delta-6 desaturase is a key enzyme for LC-PUFA biosynthesis using FA from the C16, C18, C20, C22 and C24 classes, and increased PUFA levels in the diet has been shown to decrease mammary expression of *D6D* [41]. Likewise, because the daily dietary PUFA intake was lowest in sows fed CO but highest in sows fed SO, the CO fed sows had greater mammary expression of *D6D* as compared with sows fed the SO diet. The expression of *α-LA* is a needed co-factor for the rate-limiting enzyme catalyzing synthesis of milk lactose [42] and hence is also related to sow milk yield due to the osmotic properties of lactose. No evidence for differences in mammary *α-LA* expression across diets was found and it was consistent with the similar daily milk yield and milk lactose output, which determined the estimated amount of glucose used for de novo fat synthesis in the assumptions for methods 1 and 2. The above results indicated that a low-fat diet or a diet including OFO upregulated mammary *FAS* expression to increase de novo FA synthesis, while the higher dietary PUFA in the CO diet upregulated mammary *D6D* expression to reduce milk contents of unsaturated fatty acids, suggesting a dietary fat level and FA composition dependent regulation of FA amount and profiles in sow milk.

## Conclusion

This study demonstrated that a low-fat diet reduced milk fat and energy output due to lower dietary intake of crude fat, FA, and ME, while diets containing either 4% (OFO) or 8% (FO) of fish oil reduced milk outputs of FA and FA-derived carbon due to their reduced FA intake rather than crude fat intake within high-fat diets. The difference in fat content or FA profiles in milk due to different fat supplies had no impact on piglet growth, indicating other factors are determinants of piglet growth. Among the 3 predicting models, method 1 was judged to be superior in predicting the mammary de novo fat synthesis from glucose (average to 82 g/d), showing a higher de novo synthesis of fat in sows fed low-fat or high dietary OFO diet, which was consistent with the higher mammary *FAS* gene expression in those groups. In addition, a daily intake of less than 440 g/d of digestible FA results in increased de novo fat synthesis in the mammary gland as well as increased estimates for body fat mobilization (method 3), whereas digestible intake of FA above 440 g/d results increased output in milk of dietary derived FA, thereby affecting not only FA composition but also degree of saturation in the milk. Overall, the dietary FA intake determines the conditions (i.e. proportions) among de novo fat synthesis and body fat mobilization, so that influences the profiles of FA in milk. The results of the present study underline that there are still many unresolved questions regarding the regulation of de novo fat synthesis in the mammary gland and FA profiles in milk. Although technically challenging, mass-balance studies across the mammary gland using multi-cannulated animal models in combination with tracer and omics techniques are required to further our understanding.

## Data Availability

All data of this study are included in this published article.

## Abbreviations

CO: Coconut oil
Ct: Cycle threshold
D6D: Delta-6 desaturase
DM: Dry matter
FA: Fatty acid
FAS: Fatty acid systhase
FO: Fish oil
GAPDH: Glyceraldehyde-3-phosphate dehydrogenase
GE: Gross energy
LC-MUFA: Long-chain monounsaturated fatty acids
LC-PUFA: Long-chain polyunsaturated fatty acids
MCFA: Medium-chain fatty acids
MC-SFA: Medium-chain saturated fatty acids
ME: Metabolizable energy
MUFA: Mono-unsaturated fatty acids
OA: Octanoic acid
OFO: Octanoic acid plus fish oil
PCR: Polymerase chain reaction
PUFA: Poly-unsaturated fatty acids
SO: Sunflower oil
α-LA: A-lactalbumin
